# Supplementary material for: Preparation and Study of a Simple Three-Matrix Solid Electrolyte Membrane in Air
Source: Nanomaterials (Basel). 2022 Sep 3;12(17):3069. doi: 10.3390/nano12173069 (PMC9458227; doi:10.3390/nano12173069)
Supplement: Supplementary file 1 [file nanomaterials-12-03069-s001.zip › nanomaterials-1802788-supplementary.pdf]

nanomaterials

Supplementary Materials

Preparation and Study of a Simple Three-Matrix Solid Electrolyte Membrane in Air

Xinghua Liang [1,*], Xingtao Jiang [1], Linxiao Lan [1,*], Shuaibo Zeng [2,*], Meihong Huang [3] and Dongxue Huang [1]

1 Guangxi Key Laboratory of Automobile Components and Vehicle Technology, Guangxi University of Science and Technology, Liuzhou 545006, China
2 China School of Automotive and Transportation Engineering, Guangdong Polytechnic Normal University, Guangzhou 510632, China
3 Guangdong Polytechnic of Industry and Commerce, Guangzhou 510550, China
* Correspondence: lxh18589873093@163.com (X.L.); 15506749886@163.com (L.L.); zengshuaibo@gpnu.edu.cn (S.Z.); Tel.: +86-18589873093 (X.L.); +86-18107785376 (L.L.); +86-18819264279 (S.Z.).

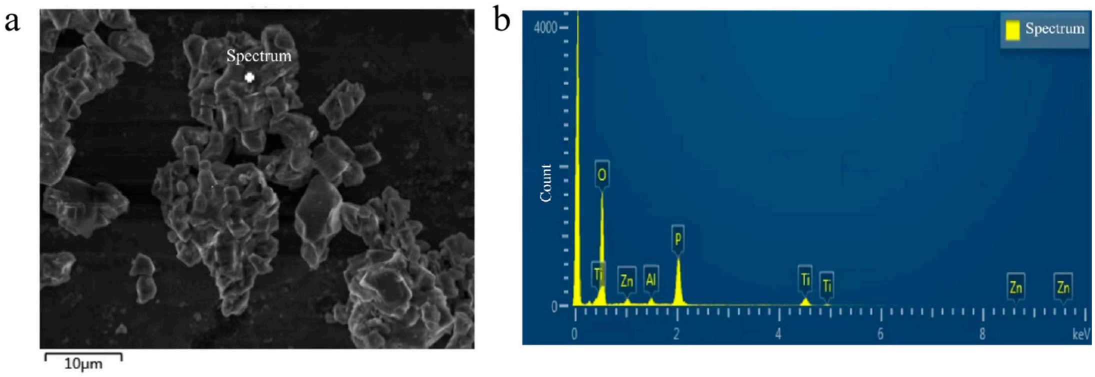

Figure S1. SEM-EDS of the LAZTP. (**a**) 10 μm size LAZTP. (**b**) Zn element content chart.

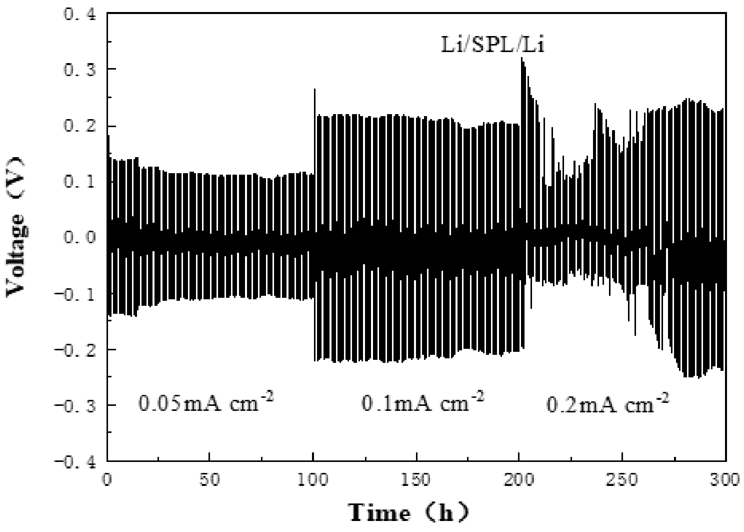

Figure S2. SPL's GCD at three different current densities.

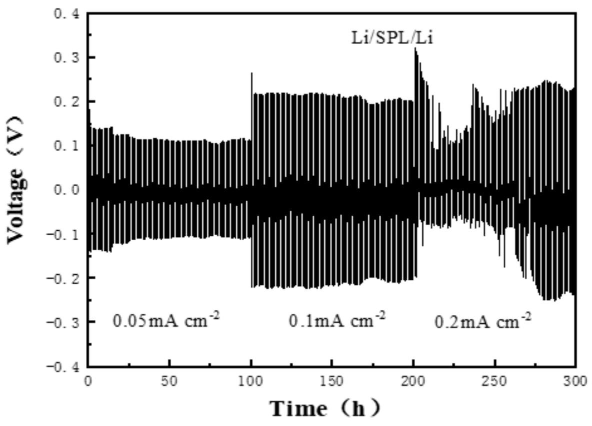

Figure S3. CV test of SPLL under different cycle rates.

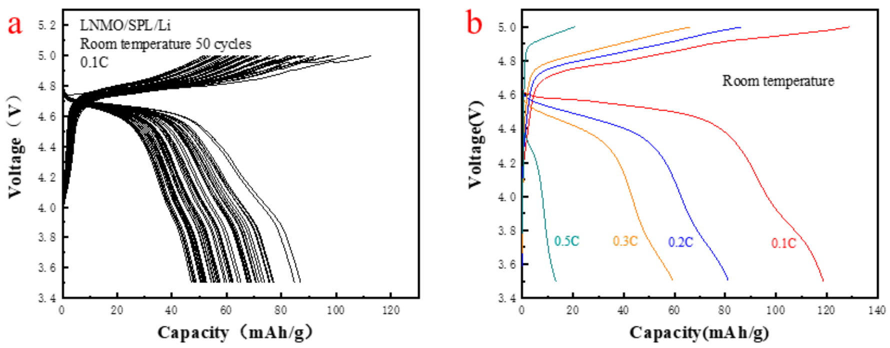

Figure S4. (**a**) 50 cycles of LNMO/SPL/Li at 0.1 C rate in the greenhouse. (**b**) The first charge and discharge of LNMO/SPL/Li at different rates.

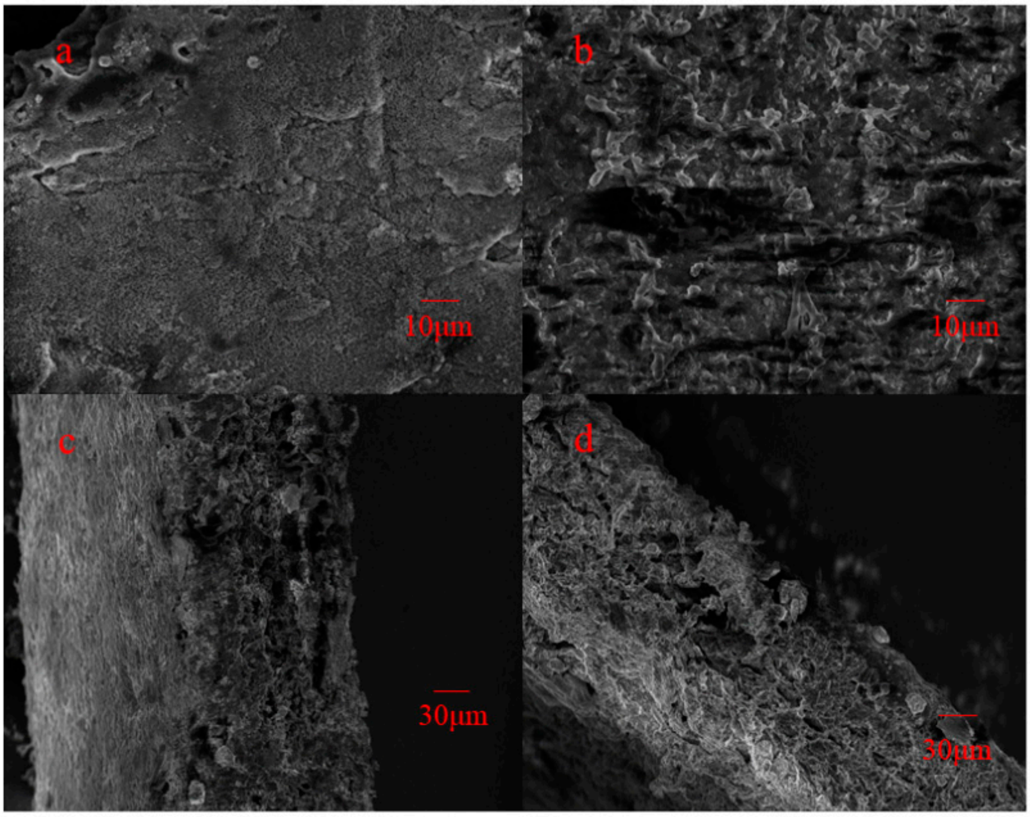

Figure S5. (**a**,**b**) Surface map of the SPLL before and after cycle. The SEM cross-sectional view of (**c**) and (**d**) SPLL cell.

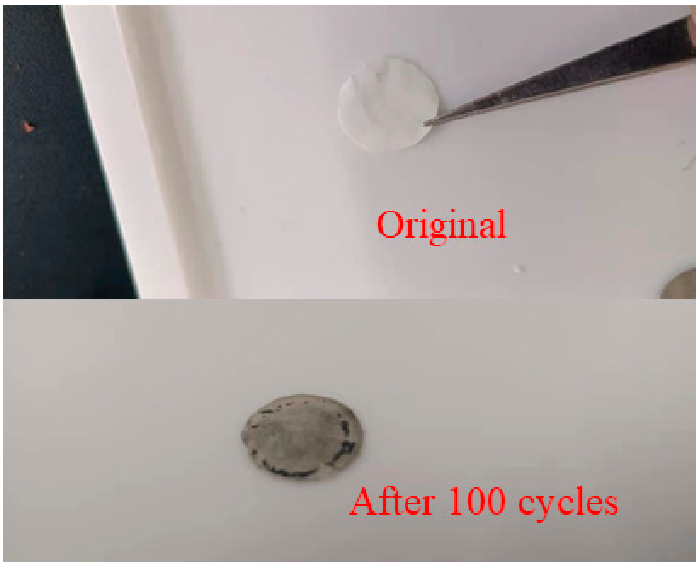

Figure S6. Physical image of the surface before and after the SPLL cycle.

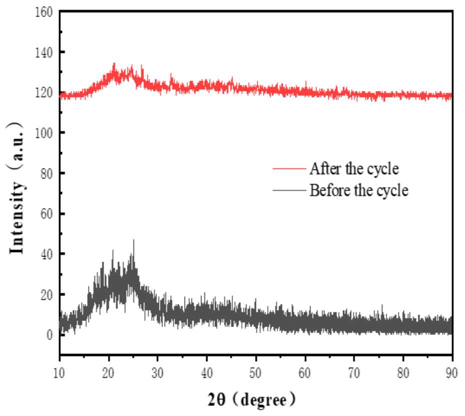

Figure S7. XRD before and after SPLL cycle.

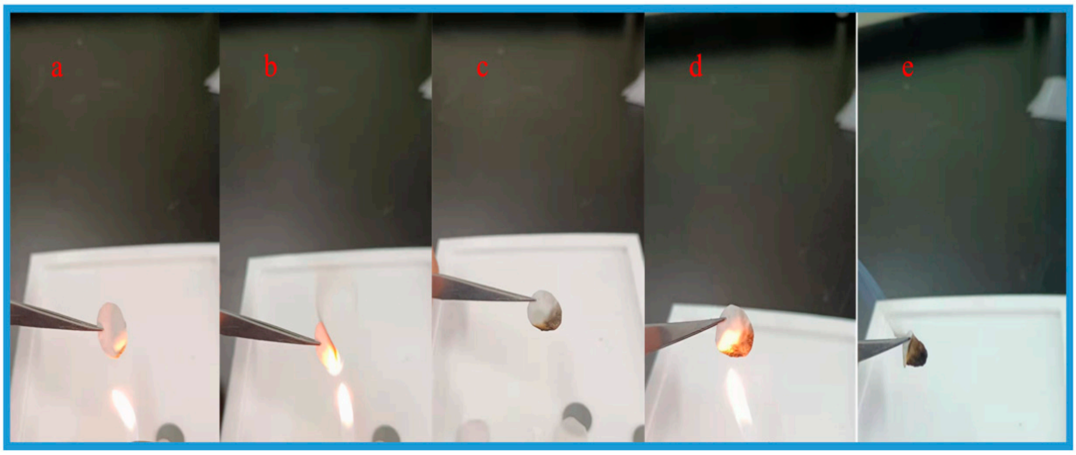

Figure S8. (**a**–**e**) Physical image of the surface before and after the SPLL cycle.
